# Transcriptome response to alkane biofuels in *Saccharomyces cerevisiae*: identification of efflux pumps involved in alkane tolerance

**DOI:** 10.1186/1754-6834-6-95

**Published:** 2013-07-05

**Authors:** Hua Ling, Binbin Chen, Aram Kang, Jong-Min Lee, Matthew Wook Chang

**Affiliations:** 1 School of Chemical and Biomedical Engineering, Nanyang Technological University, 62 Nanyang Drive, Nanyang 637459, Singapore

**Keywords:** *Saccharomyces cerevisiae*, Alkanes, Biofuels, Tolerance, Transcriptome, Efflux pumps

## Abstract

**Background:**

Hydrocarbon alkanes have been recently considered as important next-generation biofuels because microbial production of alkane biofuels was demonstrated. However, the toxicity of alkanes to microbial hosts can possibly be a bottleneck for high productivity of alkane biofuels. To tackle this toxicity issue, it is essential to understand molecular mechanisms of interactions between alkanes and microbial hosts, and to harness these mechanisms to develop microbial host strains with improved tolerance against alkanes. In this study, we aimed to improve the tolerance of *Saccharomyces cerevisiae*, a model eukaryotic host of industrial significance, to alkane biofuels by exploiting cellular mechanisms underlying alkane response.

**Results:**

To this end, we first confirmed that nonane (C9), decane (C10), and undecane (C11) were significantly toxic and accumulated in *S. cerevisiae*. Transcriptome analyses suggested that C9 and C10 induced a range of cellular mechanisms such as efflux pumps, membrane modification, radical detoxification, and energy supply. Since efflux pumps could possibly aid in alkane secretion, thereby reducing the cytotoxicity, we formed the hypothesis that those induced efflux pumps could contribute to alkane export and tolerance. In support of this hypothesis, we demonstrated the roles of the efflux pumps Snq2p and Pdr5p in reducing intracellular levels of C10 and C11, as well as enhancing tolerance levels against C10 and C11. This result provided the evidence that Snq2p and Pdr5p were associated with alkane export and tolerance in *S. cerevisiae*.

**Conclusions:**

Here, we investigated the cellular mechanisms of *S. cerevisiae* response to alkane biofuels at a systems level through transcriptome analyses. Based on these mechanisms, we identified efflux pumps involved in alkane export and tolerance in *S. cerevisiae*. We believe that the results here provide valuable insights into designing microbial engineering strategies to improve cellular tolerance for highly efficient alkane biofuel production.

## Background

Next-generation biofuels, such as long-chain alcohols, and fatty-acid- and isoprenoid-derived fuels, offer advantages of high energy density, low freezing point, and compatibility with the existing fuel storage and distribution infrastructure [1-4]. Recently, hydrocarbon alkanes, main components of fossil fuels, have been considered as important next-generation biofuels because their microbial production was demonstrated. Schirmer et al. converted intermediates of fatty acid metabolism to alkanes in *Escherichia coli* by introducing two enzymes involved in an alkane biosynthesis pathway from cyanobacteria [5]. Bernard et al. identified *Arabidopsis* alkane synthesis enzymatic components and reconstituted plant alkane biosynthesis in yeast [6].

Despite these successes in microbial alkane production, the toxicity of alkanes to microbial hosts can eventually be a bottleneck for high productivity. To overcome this toxicity issue, it is imperative to develop engineering strategies to improve microbial tolerance against biofuel alkanes, which requires a clear understanding of molecular mechanisms of interaction between microbial hosts and alkanes. Physiologically, hydrocarbon accumulation in cell membrane causes loss of membrane integrity and function, which ultimately leads to cell death [7]. In response, cells protect themselves against the toxicity of hydrocarbons by ordering the lipid bilayer to modify lipopolysaccharide (LPS) and cell wall/S-layer hydrophobicity, as well as activating the excretion by energy-consuming transport systems [8]. Despite the aforementioned studies, which primarily focused on physiological or cytological effects, there is lack of understanding of the molecular mechanisms of interactions between microbes and alkanes especially at a systems level.

Toxicogenomics, which combines genomics and toxicology, is useful for identification of toxicants and their putative mechanisms of action at a systems level. Recently, toxicogenomics has been applied to elucidate mechanisms underlying environment stresses and chemical toxicity to microorganisms [9,10]. For instance, Chang et al. studied the toxicogenomic response of pathogens to antimicrobials by using microarray-based transcriptome analyses [11]. Carvalho et al. studied transcriptomic response in marine diatom *Thalassiosira pseudonana* exposed to benzo[a]pyrene [12]. Notably, toxicogenomics has also offered an effective means to study the molecular mechanisms of cell response to organic solvents. For example, based on genome-wide microarray analyses, our group previously identified and reconstituted genetic regulatory networks to improve the tolerance of *E. coli* against isooctane [13]. In *Saccharomyces cerevisiae* strain KK-211, genes involved in tolerance to organic solvents were successfully identified based on transcriptome analyses [14,15]. Note that *S. cerevisiae*, an established and widely used eukaryotic model for molecular and cellular biology studies, has been used as an experimental model in toxicogenomics [16-18]. In this light, this study aimed to (i) investigate molecular mechanisms underlying cellular response to alkanes at the systems level and (ii) improve cellular tolerance based on these mechanisms in *S. cerevisiae*, a model eukaryotic host of industrial significance. Briefly, we showed that nonane (C9) and decane (C10) induced cellular mechanisms associated with efflux pumps, membrane modification, radical detoxification, and energy supply. More importantly, based on these cellular mechanisms, we demonstrated that efflux pumps Snq2p and Pdr5p played roles in reducing intracellular levels of C10 and undecane (C11), and enhancing tolerance levels against C10 and C11. Given the aforementioned recent reports on microbial alkane production [5,19], we envision that the engineering strategy established in this study can readily be extended to develop robust microbial hosts for alkane biofuels production.

## Results and discussion

### Toxicity and intracellular accumulation of alkanes

**Figure 1:**
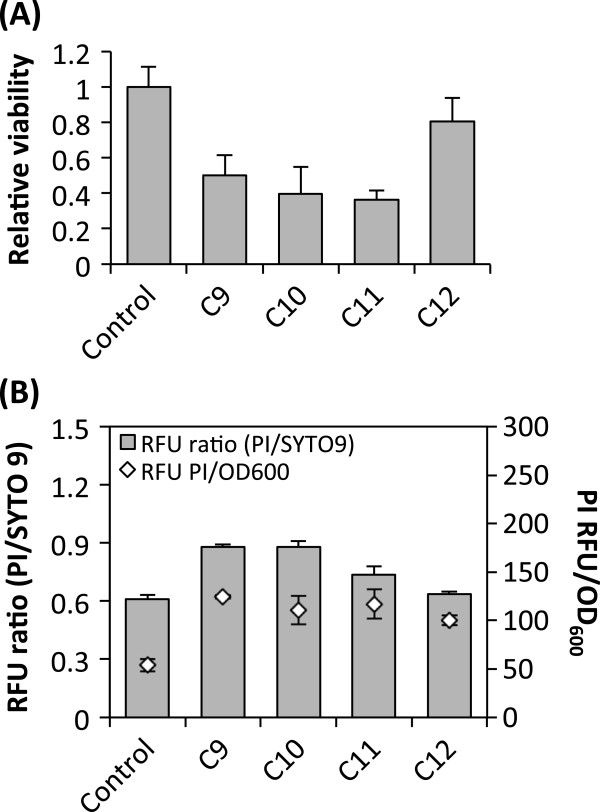
**Viability and membrane permeability of *S. cerevisiae *cells under exposure to C9-C12.** The viability **(A)** and membrane permeability **(B)** were measured upon exposure to alkanes (2% each) for 48 h. RFU, relative fluorescence unit. Error bars represent standard deviations from four biological replicates.

**Figure 2:**
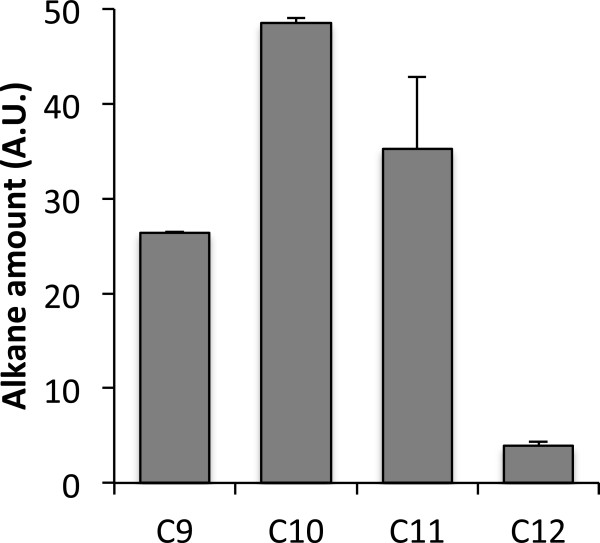
**GC analyses of intracellular alkanes in *S. cerevisiae*.** Cells were exposed to 2% of each n-alkane for 48 h. Intracellular alkanes were measured by GC and normalized to the total protein amount and the internal standard n-tridecane. A.U., arbitrary unit. Error bars represent standard deviations from three biological replicates.

We first examined the toxicity of nonane (C9), decane (C10), undecane (C11), and dodecane (C12). In this work, we primarily focused on C9-C12 because our preliminary studies had indicated low cytotoxicity of C13 and longer alkanes. *S. cerevisiae* BY4741 cells were incubated in the medium containing each alkane (2% [v/v]). Figure 1A shows that cell viability was decreased upon exposure to C9-C12. In particular, treatment with C9-C11 decreased cell viability by about 50%. Based on this result, we hypothesized that C9-C12 might affect cell membrane permeability or integrity. Figure 1B shows that the intensity of red fluorescent signal of propidium iodide (PI) and the ratio of the fluorescent signals (PI/SYTO9) were increased upon exposure to C9-C12. If more PI molecules penetrate into cell wall/membrane and stain nucleic acids, PI intensities and PI/SYTO 9 ratios become higher. Therefore, this result suggests that C9-C11 increased cell membrane permeability. Among these alkanes, C9 and C10 resulted in the highest PI intensities and PI/SYTO9 ratios, which indicates the highest membrane permeability. To determine whether this increased membrane permeability was contributed by damage on cell surface, cell surface was examined using Field Emission Scanning Electron Microscopy (FESEM). As shown in Additional file 1: Figure S1, damage was not apparent on cell surface upon exposure to 2% C9, which is in agreement with de Smet and coworkers’ study [20]. Based on this result, we hypothesized that alkane toxicity might be attributed at least partly to intracellular accumulation. GC analyses show that a peak corresponding to each alkane was detected from alkanes-treated cells, while control cells without alkanes exhibited a negative signal (Additional file 2: Figure S2). To measure intracellular alkane amounts, we normalized GC peak areas to the internal standard and the total protein amount. Among C9 to C12, C10 showed the highest intracellular level, followed by C9 and C11, and C12 level was the lowest which was 90% lower than C10 level (Figure 2). These results indicate that alkanes were accumulated in cells, which likely contributed to alkane toxicity. According to Inoue and Kawamoto [21,22], toxicity of a compound is related to coefficient of octanol-water partition (log *P*_*ow*_). Gill and coworkers reported that higher aliphatic chain alkanes tend to show lower toxicity [23]. In our study, C12 (log *P*_*ow*_ 7.0) showed lower toxicity than C9 to C11 (log *P*_*ow*_ <6.0), which could be partially due to low accumulation of C12 in *S. cerevisiae*.

### Genome-wide gene expression profiles upon exposure to biofuel alkanes

To understand cellular mechanisms underlying *S. cerevisiae* response to C9-12, transcriptome profiles were generated using whole-genome *S. cerevisiae* microarrays. Significantly regulated genes (fold changes ≥ 2.0, and p values < 0.05) are summarized in Additional file 3. The data discussed in this publication have been deposited in NCBI’s Gene Expression Omnibus and are accessible through GEO Series accession number GSE38653. In addition, quantitative PCR (qPCR) was carried out to validate the microarray data (Additional file 3).

Our transcriptome analysis indicated that 526 (C10 for 24 h), 692 (C9 for 48 h), 428 (C10 for 48 h), and 119 (C11 for 48 h) genes were differentially regulated, while 5 (24 h) and 8 (48 h) genes were regulated by C12 (Additional file 3). Functional analysis of those genes suggested that C11 primarily regulated genes related to heat shock response and sugar transport, whereas C9 and C10 induced a range of similar groups of genes. Therefore, to look into cellular mechanisms commonly stimulated by alkanes, we analyzed the genes regulated by both C9 and C10. 147 genes were significantly regulated by both C9 and C10. Among the 147 genes, 105 genes were induced by both C9 and C10, whereas 42 genes were repressed by both C9 and C10 (except for *COS1*2 gene which was down-regulated by C10 but up-regulated by C9). To further understand gene functions, we functionally classified the aforementioned regulated genes using MIPS tools (http://funspec.med.utoronto.ca/) [24]. Primary functional classes of the regulated genes were (i) efflux pumps, (ii) stress response, (iii) fatty acid, lipid, and derivatives biosynthesis, and (iv) hexose transport, ergosterol biosynthesis, and thiamine biosynthesis (Table 1). The genes in these classes were mostly induced by C9 and C10.

**Table 1:** MIPS functional classification of genes differentially regulated by both C9 and C10

**Functional category**	**k**	**f**
***Up-regulated***		
fatty acid metabolism	5	24
phospholipid metabolism	7	68
oxidative stress response	6	55
tetracyclic and pentacyclic triterpenes	5	36
cellular import	7	90
lipid/fatty acid transport	5	44
ABC transporters	4	28
lipid, fatty acid and isoprenoid metabolism	8	133
degradation of glutamate	2	4
metabolism of amino acid-derived secondary products	2	4
NAD/NADP binding	4	36
C-compound and carbohydrate metabolism	10	223
heat shock response	3	20
chemical agent resistance	3	22
S-adenosyl-methionine-homocysteine cycle	2	7
pH stress response	2	8
oxidation of fatty acids	2	9
transport ATPases	4	53
***Down-regulated***		
biosynthesis of thiamine	6	110
transport facilities	5	87
degradation of lysine	2	5
degradation of glycine	2	6
transport routes	2	11
metabolism of thiamine	3	43
tetrahydrofolate-dependent C-1-transfer	2	13

Note: functional classification was generated by the online program FunSpec (http://funspec.med.utoronto.ca/) with p-value cut-off of 0.01. k, number of genes from the input (105 up-regulated genes, and 42 down-regulated genes) in the given category, f, number of genes total in the given category.

First of all, notably, four plasma membrane efflux pump genes *YOR1*, *SNQ2*, *PDR5,* and *PDR15* were significantly induced by alkane stress. Plasma membrane efflux pumps recognize and extrude a large spectrum of functionally and structurally unrelated drugs such as oligomycin, mycotoxins, and anticancer drugs [25-28]. That is, when cytotoxic compounds cross the plasma membrane to enter cells, a number of efflux pumps help to protect cells from unwanted or damaging xenobiotics. According to Ernst and coworkers [29], efflux pumps serve as a defense line mediating pleiotropic drug resistance (PDR). Therefore, the result that efflux pump genes were significantly induced here suggested that they might also provide protection to the cells against alkanes.

Other than efflux pump genes, genes involved in stress response for radical detoxification, fatty acid and lipid synthesis, and hexose transport were also induced by alkanes. For example, *GRE* genes associated with multiple stresses [30] and heat shock protein gene *HSP12* were induced up to 16-fold, and fatty acid synthase genes *FAS1* and *FAS2* were induced over 4-fold. Acetyl-CoA carboxylase gene *ACC1* was up-regulated about 6-fold. Acc1p is a biotin-containing enzyme that participates in fatty acid synthesis by converting acetyl-CoA to malonyl-CoA, which is a rate-limiting step in the fatty acid synthesis pathway [31]. In addition, *INO1* gene was induced over 50-fold, which is associated with synthesis of phosphatidylinositol by using glucose-6-P and CDP-DAG as precursors [32].

**Figure 3:**
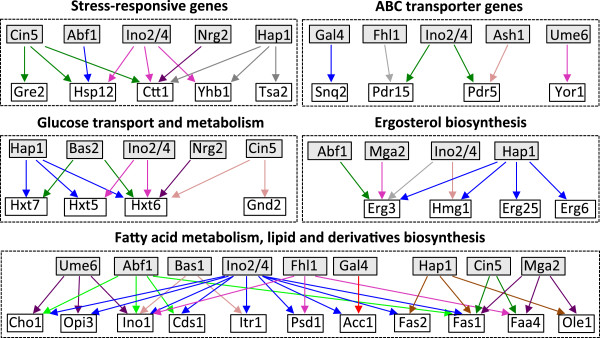
**The predicted transcription regulatory network.** Regulation matrices were generated by YEASTRACT (http://www.yeastract.com/) based on the documented regulations, with our transcription factors as regulator candidates and regulated genes as target candidates. Transcription factors are shown in a grey box, and target genes are in an open box. Genes regulated by the same transcription factor in each group are shown with arrows in the same color.

Upon alkanes exposure, six transcription factor genes, *ASH1*, *GAL4*, *NRG2*, *MGA2*, *YPR015C,* and *THI2*, were differentially regulated. Because changes in gene expression levels of transcription factors frequently do not correlate with changes in their regulatory activities, to further identify transcription factors with perturbed regulatory activities under alkane exposure, we analyzed our transcriptome data using Network Component Analysis (NCA). NCA is an algorithm that calculates relative activities of transcription factors from gene expression data [13]. NCA results suggested that regulatory activities of nine transcription factors were changed upon exposure to C9 (48 h) and C10 (24 h, and 48 h) (Table 2). Among the nine transcription factor genes identified from NCA, the activities of six transcription factors were increased, and the activities of three were decreased. To further understand regulatory relationships between transcription factors and the genes involved in C9 and C10 response, a regulation matrix was generated by YEASTRACT (http://www.yeastract.com/) [33]. In the generated transcription networks (Figure 3), *INO2* showed regulation of genes associated with efflux pumps, stress response, lipid metabolism and ergosterol biosynthesis. Hereby, we predicted that *INO2* would most likely be one of the key transcription factors that regulate gene networks in response to alkanes exposure in *S. cerevisiae*. Further, the three repressed transcription factors (i.e. *CIN5*, *FHL1*, and *HAP1*) likely up-regulate their target genes such as *GRE2*, *GND2*, and *ERG6* (Figure 3). Therefore, there is a possibility that the aforementioned transcription factors induce genes associated with efflux pumps, stress response, and lipid metabolism in *S. cerevisiae* upon exposure to both C9 and C10.

**Table 2:** Transcription factors with activity changes, predicted by Network Component Analysis (NCA)

**Transcription factors**		**Regulatory activity**		
		**C10-24 h**	**C10-48 h**	**C9-48 h**
YKL112W	*ABF1*	+3	+3	+3
YKR099W	*BAS1*	+3	+3	+4
YDR123C	*INO2*	+12	+18	+19
YOL108C	*INO4*	+3	+4	+3
YGL162W	*SUT1*	+4	+5	+5
YDR207C	*UME6*	+6	+4	+4
YOR028C	*CIN5*	−2	−3	−10
YPR104C	*FHL1*	−3	−4	−8
YLR256W	*HAP1*	−3	−3	−7

Note: C10-24 h, cells exposed to C10 for 24 h compared to cells without alkane exposure for 24 h, C9- or C10-48 h, cells exposed to C9 or C10 for 48 h compared to cells without alkane exposure for 48 h. +, upregulation, -, downregulation.

**Figure 4:**
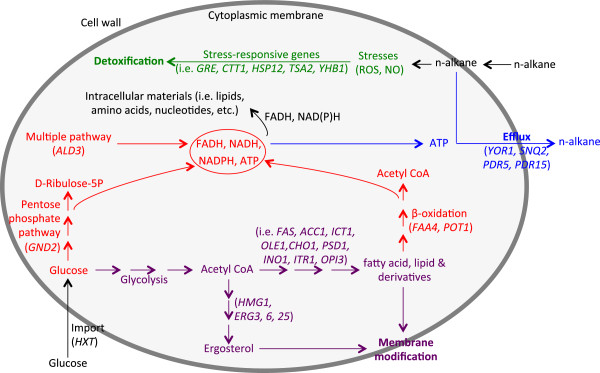
**Schematic representation of hypothesized mechanisms of cellular response to alkanes in *S. cerevisiae*.** The mechanisms include alkane efflux (blue), detoxification of radicals (green), energy supply (red), and membrane modification (purple).

Based on our transcriptome data, and the functions and physiological roles of the regulated genes, we hypothesized that *S. cerevisiae* responded to alkanes stresses through integrative mechanisms (depicted in Figure 4). First, efflux pump genes (*YOR1, SNQ2, PDR5*, and *PDR15*) are induced to export intracellular alkanes to reduce alkane toxicity. Second, alkanes exposure generates radicals and induces stress-responsive genes towards radical detoxification. Alkane molecules penetrate cell wall, disperse into cytoplasmic membrane, and generate radicals (e.g. reactive oxygen species (ROS), and NO), while stress-responsive genes (*GRE1*, *GRE2*, *CTT1*, *HSP12*, *TSA2*, and *YHB1*) are induced to detoxify radicals including ROS. Notably, in line with this hypothesis, we observed that ROS levels were increased by 110% and 78% in *S. cerevisiae* upon exposure to C9 and C10, respectively (Additional file 3). Such increase in ROS levels is most likely correlated with alkanes toxicity. Third, more energy sources are generated through acceleration of glucose and fatty acid metabolism. Hexose transport genes (*HXT5, HXT6*, and *HXT7*) are induced to increase glucose import. Thereafter, energy sources (FADH, NADH, NADPH, and ATP) are enriched through enhanced glucose metabolism involving pentose phosphate pathway *(GND2*)*,* β-oxidation of fatty acids *(FAA4*, and *POT1*), and *ALD3*-involved pathways. ATP may be used for alkanes export by efflux pumps. Fourth, the increase in glucose import and consumption aids in modifying membrane components (i.e. lipid, and ergosterol), thereby helping cells to adapt to alkanes exposure. Enriched glucose inside the cells may lead to the synthesis of more fatty acids, lipids, and derivatives (i.e. phospholipid, glycerophospholipid, phosphatidylinositol, and phosphatidylcholine), contributed to by such induced genes as *FAS1, FAS2*, *ACC1*, *ICT1*, *OLE1*, *CHO1*, *PSD1*, *INO1*, *ITR1*, and *OPI3*. Further, ergosterol biosynthesis is enhanced by the four induced genes *HMG1*, *EGR3, ERG6*, and *ERG25*. Such a change in the expression of genes involved in lipid and ergosteols metabolism may lead to membrane modification, possibly facilitating adaptation to alkanes exposure.

### Overexpression of efflux pumps leads to improved tolerance to biofuel alkanes

Among the aforementioned mechanisms, we were interested in further identifying roles of plasma membrane efflux pumps in alkane tolerance and export because (i) plasma membrane efflux pumps are the first line of defense against drugs or organics, and (ii) microbial efflux pumps reportedly serve as a direct mechanism to improve biofuel tolerance [4,34-37] as well as productivity [4]. In fungi, efflux pumps, especially ATP-binding cassette (ABC) efflux pumps (e.g. Pdr5p and Snq2p in *S. cerevisiae*, Cdr1p and Cdr2p in *Candida albicans*), which contribute to multidrug efflux resistance, have been most extensively studied [25-28,38]. In our study, all the four efflux pumps induced by alkanes are ABC transporters, and considered as major pleiotropic drug transporters that comprise a PDR network in yeast [39]. Among them, Snq2p, Pdr5p, and Pdr15p belong to a pleiotropic drug resistance (PDR) protein subfamily, and Yor1p belongs to a multidrug resistance-associated protein (MRP) subfamily. Their protein architecture contains multiple transmembrane domains (TMDs) that bind chemical compound and provide a translocation channel, and nucleotide-binding domains (NBDs) that couple ATP hydrolysis to substrate transport. Given that the induced ABC efflux pumps play roles in reducing intracellular alkane accumulation, tolerance of *S. cerevisiae* cells against alkanes could be improved. Hence, we formed a hypothesis that the significantly induced efflux pumps might also help cells to reduce the intracellular alkane accumulation and thus, provide protection to cells against alkanes.

**Figure 5:**
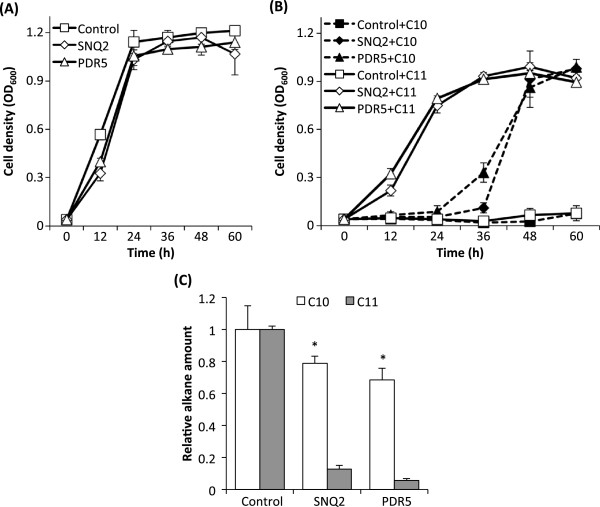
**Growth of triple knock-out mutants expressing efflux pumps and their intracellular alkane levels under alkane exposure.** The triple mutant BYL251K (*yor1∆ snq2∆ pdr5∆*) expressing Snq2p and Pdr5p, respectively, were grown in induction media without alkanes **(A)** or with 2% C10 or 5% C11 **(B)**. OD_600_ values represent cell density. After 24 h exposure, intracellular C10 and C11 levels were measured by GC and normalized to the total protein amount and the internal standard C12 **(C)**. Alkane amount in control cells was set as 1. Control, the triple mutant BYL251K with pYES2. Error bars represent standard derivations of at least three biological replicates. *, statistically significant difference (Student *t*-test, p < 0.05) compared to cells with pYES2.

To identify roles of each of the induced efflux pumps in reducing intracellular alkane levels and improving alkane tolerance levels, we constructed triple knock-out mutants of efflux pumps, BYL251K (*yor1∆ snq2∆ pdr5∆*) and BYL2515K (*snq2∆ pdr5∆ pdr15∆*). Subsequently, we cloned the four efflux pump genes (*YOR1*, *SNQ2*, *PDR5*, and *PDR15*) into plasmid pYES2 respectively, and transformed recombinant plasmids into the triple mutants. Under control of the galactose-inducible promoter P_GAL1_ and exposure to alkanes, the aforementioned efflux pump genes (*YOR1*, *SNQ2*, and *PDR5*) were expressed in the triple mutant BYL251K respectively, and *PDR15* was expressed in the triple mutant BYL2515K. Cell tolerance and intracellular alkane levels were then analyzed (Figure 5, and Additional file 4: Figure S3). Figure 5A shows that there was similar growth among the BYL251K cells with pYES2 (control) and with *SNQ2* or *PDR5* in an alkane-free induction medium. Upon exposure to C10, cell growth of the triple mutant BYL251K expressing each of Snq2p and Pdr5p was significantly enhanced, compared to BYL251K with the plasmid pYES2 (Figure 5B). This result suggested that expression of Snq2p and Pdr5p, respectively, increased cellular tolerance to C10. Furthermore, Figure 5C shows that upon 24 h exposure to C10, intracellular C10 amount was lowered by 25% and 33% in the triple mutant BYL251K expressing either Snq2p or Pdr5p. This result indicated a crucial role of Pdr5p and Snq2p in reducing the intracellular C10 levels, consistent with the hypothesis on the role of efflux pumps in improving alkane tolerance as mentioned above. Furthermore, we tested growth of cells expressing Pdr5p and Snq2, respectively, under 5% C11. In support of our hypothesis, the triple knock-out mutant BYL251K expressing the efflux pump Snq2p or Pdr5p showed much higher cell density than the control cells, even though C11 did not induce expression of those efflux pumps according to our microarray data. This result suggested that tolerance to C11 was significantly improved by the efflux pumps Snq2p and Pdr5p. Further, Figure 5C shows that intracellular C11 levels were lowered by 87.4% and 94.4% in the triple mutant BYL251K expressing Snq2p and Pdr5p, respectively. This result indicated a crucial role of Pdr5p and Snq2p in reducing the intracellular C11 levels, consistent with the aforementioned hypothesis on the role of efflux pumps in improving the tolerance to C11. Note that the Resistance-Nodulation-Division (RND) efflux pumps such as AcrAB-TolC pump, Mex pumps, Ttg pumps from Gram-negative bacteria have been previously studied as an effort to improve tolerance to biofuels in bacteria [34,35,37,40]. However, the RND pumps utilize proton or sodium gradients as a source of energy [41,42] instead of ATP by ABC efflux pumps. This suggests that yeast and gram-negative bacteria employ different types of efflux pumps for improving the biofuel tolerance.

However, even though the efflux pump genes (*YOR1*, *SNQ2*, *PDR5,* and *PDR15*) were all induced by both C9 and C10 according to our microarray data (Additional file 3), there was no improvement in tolerance against C9 by complementation of each of the four efflux pumps. In addition, there was no improvement in tolerance against C10 and C11 by complementation of Yor1p or Pdr15p. This outcome was probably due to variance in substrate binding efficacy of various efflux pumps, and/or the involvement of such mechanisms as stress response and membrane modification, in addition to efflux pumps, in cellular tolerance to C9. Engineering approaches such as site-mutation, evolutionary engineering, and gene shuffling can be employed to improve substrate binding efficacy [10,43-45]. Other than the indigenous efflux pumps in *S. cerevisiae*, efflux pumps from other microbes can also be introduced to improve tolerance against biofuel alkanes. In addition, future studies could include use of other cellular mechanisms besides efflux pumps as an effort to improve alkane tolerance.

## Conclusions

To our knowledge, this is the first report providing the evidence of the direct linkage between alkane tolerance and indigenous efflux pumps, and of being able to develop alkane-tolerant *S. cerevisiae* by directly harnessing indigenous efflux pumps. Herein, we first confirmed that C9, C10, and C11 were significantly toxic and accumulated in *S. cerevisiae*. Transcriptome analyses suggested that C9 and C10 induced a range of cellular mechanisms such as efflux pumps, membrane modification, radical detoxification, and energy supply. Since efflux pumps could possibly aid in alkane export, thereby reducing the cytotoxicity, we formed the hypothesis that those induced efflux pumps could contribute to alkane tolerance. In support of this hypothesis, we demonstrated the roles of the efflux pumps Snq2p and Pdr5p in reducing the intracellular levels of C10 and C11, and enhancing the tolerance levels against C10 and C11. This result provided the evidence that Snq2p and Pdr5p were associated with alkane export and tolerance in S. *cerevisiae*. We believe that the results here provide valuable insights into designing microbial engineering strategies to improve cellular tolerance for highly efficient alkane biofuel production.

## Methods

### Strains, chemicals and plasmids

*S. cerevisiae* BY4741 (MATa; his3 ∆1; leu2∆0; met15∆0; ura3∆0) was obtained from ATCC (American Type Culture Collection), and gene deletion mutants were constructed in this study (Additional file 3). Yeast extract and peptone were obtained from BD (NJ, USA). Alkanes and other chemicals were obtained from Sigma-Aldrich unless specifically mentioned. pUG6 and pSH47 were used for gene deletion and marker rescue in yeast cells. Plasmid pYES2 (Invitrogen, Grand Island, NY, USA) was used for gene expression in yeast cells. *E. coli* Top10 was used for gene cloning.

### Growth conditions

*S. cerevisiae* strains were grown in YPD medium (Yeast extract 10 g/l, Peptone 20 g/l, and D-Glucose 20 g/l), minimum medium (Yeast Nitrogen Base 6.7 g/l, D-Glucose 20 g/l, L-Leucine 0.2 g/l, Uracil 0.1 g/l, 2% n-alkanes; Yeast Nitrogen Base 6.7 g/l, D-Glucose 20 g/l, Yeast Synthetic Drop-out Medium Supplements-Ura^-^ 1.92 g/l), or induction medium (Yeast Nitrogen Base 6.7 g/l, Raffinose 10 g/l, Galactose 20 g/l, and Yeast Synthetic Drop-out Medium Supplements-Ura^-^ 1.92 g/l) at appropriate temperatures. *E. coli* cells were grown in LB broth at 37°C. Geneticin G418 (PAA Laboratories GmbH, AT) or Ampicillin was used at 200 μg/ml and 100 μg/ml, respectively.

### Cell viability, membrane integrity and reactive oxygen species (ROS)

*S. cerevisiae* BY4741 cells were grown overnight in YPD, diluted into minimal medium with amino acids which contains each of 2% alkanes (C9-C12) with an initial OD_600_ of 0.2, and followed by shaking incubation at 28°C, and viable cells measurement by CFU (Colony Forming Unit) counting. To investigate whether these alkanes resulted in membrane damage, membrane permeability was analyzed by cell staining. After exposure to alkanes, cells were stained with fluorescent nucleic acid stains SYTO 9 and propidium iodide (PI) (Invitrogen) that only penetrates cells with damaged membrane. Finally, signal intensity in cells was measured using Synergy HT multi-mode microplate reader (Biotek, VT, USA) at wavelength of 485/20 nm (excitation), 528/20 nm (SYTO 9 emission), and 645/40 nm (PI emission). To investigate reactive oxygen species (ROS) generation, alkane-treated cells were collected and stained by CellROX® Green Reagent (Life Technologies). Fluorescence signals were measured by TECAN Infinite 200 microplate reader at wavelength of 485 nm (excitation)/535 nm (emission) and normalized to cell density (OD_600_).

### Alkane extraction and detection

Alkanes were extracted by chloroform-methanol extraction method [46] with modifications. Firstly, yeast cells were collected and washed with 50 mM Tris.Cl (pH 7.4) then resuspended in 0.4 ml chloroform/methanol mixture (v/v: 2/1). Subsequently, 0.3 g acid-washed glass beads (425-600 μm, Sigma-Aldrich) were added into each sample and cells were lysed using FastPrep-24 (MP Biomedicals, Solon, OH, USA). Then, after the lysate was centrifuged at 4°C at maximum speed for 10 min, an appropriate volume of chloroform and 50 mM Tris.Cl were added to the supernatant for extraction. Finally, the chloroform phase with alkanes was transferred into glass vials for Gas Chromatography (GC) analysis. This was performed using Agilent GC 7890A system (Agilent Technologies, Santa Clara, CA, USA) under the following conditions. With Agilent HP-5 GC column, oven temperature was started at 40°C for 30 sec, and was ramped from 40°C to 220°C at 40°C/min, and FID detector temperature remained at 275°C. A mixture of n-alkanes (10 ppm each) was used as the standard for GC analysis.

### RNA preparation, transcriptome analysis, and quantitative PCR

Total RNA was extracted with lyticase for cell wall disruption using RNeasy Mini Kit (Qiagen, Hilden, Germany). Total RNA concentration and integrity were measured using NanoDrop (Thermo Scientific, Wilmington, DE, USA) and Agilent Bioanalyzer 2100, respectively. Subsequently, 200 ng of the qualified total RNA was transcribed and labeled using Agilent RNA Spike-in Kit and Low Input Quick Amp Labeling Kit (two colors). The Cy3- or Cy5-labeled cRNA was hybridized onto Yeast (V2) Gene Expression Microarray (8 × 15 K) at 65°C for 17 h, using Agilent GE Hybridization Kit. Thereafter, the microarray slide was scanned using Agilent Microarray Scanner G2505B. The two-color scanning data were extracted using Agilent Feature Extraction software (v10.5) and analyzed using GeneSpring GX 11. From two biological replicates, differentially expressed genes were selected based on their p values (<0.05, *t*-test) and fold changes (FC) in n-alkane-treated cells in comparison to control cells (≥2.0).

To validate the microarray data, quantitative PCR (qPCR) was performed with primers targeting the selected genes (Additional file 3) and SsoFast™ EvaGreen® Supermix using iQ5 real-time PCR detection system (Bio-Rad). The qPCR data analysis was conducted with *ACT1* as a reference gene using Bio-Rad iQ5 optical system software.

### Network component analysis (NCA)

The Network Component Analysis is an algorithm that calculates relative activities of transcription factors from gene expression data [13]. In brief, a matrix of gene expression data ([E]), which contain genes in rows and experimental conditions in columns, are prepared for NCA analysis. Subsequently, the [E] matrix is decomposed into two matrices, a connectivity strength (CS) matrix ([A]) with transcription factors (columns) and genes (rows) and a transcription factor activity (TFA) matrix ([P]) with transcription factors (rows) an experimental conditions (columns). In our study, to perform NCA analysis, first, genes in the gene expression data matrix ([E]) were built from 147 genes (fold changes ≥ 2.0, and p values < 0.05), up- or down-regulated under the three conditions (C10 vs. control at 24 h and 48 h, and C9 vs. control at 48 h); second, the connectivity strength (CS) matrix [A] from Lee and coworkers [47] was used to primarily define connectivity relations between the genes and the transcription factors; third, the final TFA matrix [P] was deduced by NCA.

### Yeast gene deletion

Gene deletion was carried out as previously described [48]. Briefly, *loxP-Kan-loxP* gene disruption cassettes were amplified using PCR with primers as shown in Additional file 3 and pUG6 as the template, which were then purified using QIAquick Gel Extraction Kit (Qiagen). The purified cassettes were used for yeast transformation as previously described. The colonies were screened on YPD plates containing 200 μg/ml G418 and confirmed by PCR. To perform marker rescue, pSH47 was transformed into mutants, selected on minimal medium (Ura^-^) plates, and followed by Cre/loxP-mediated marker removal in induction medium. Finally, selection for the loss of marker was performed in YPD containing 1 mg/ml 5-fluoro-orotic acid (Thermo Scientific).

### Plasmid construction and analyses of yeast strains overexpressing efflux pump genes

The gene specific primers (Additional file 3) and iProof High-Fidelity DNA Polymerase (Bio-Rad) were employed to amplify target genes by PCR with *S. cerevisiae* BY4741 genomic DNA as a template. DNA fragments were purified and cloned into an expression vector pYES2. The recombinant plasmids were prepared using QIAprep Miniprep Kit (Qiagen), transformed into *S. cerevisiae* mutants by electroporation [49], and followed by selection on Geneticin-containing minimal medium plates. To investigate effects of mutants expressing efflux pumps on cell growth and alkane accumulation, cells harboring a recombinant plasmid were grown in an induction medium plus alkanes. Subsequently, optical density of cells at 600 nm (OD_600_) and intracellular alkanes were measured.

## Abbreviations

NCA: Network component analysis; C9: n-nonane; C10: n-decane; C11: n-undecane; C12: n-dodecane.

## Competing interests

The authors declare that they have no competing interests.

## Authors’ contributions

HL and MWC conceived the project, designed the experiments, and wrote the manuscript. HL and BC conducted the experiments. HL, BC, AK and JL analyzed the data. MWC supervised the project. All authors read and approved the final manuscript.

## Supplementary Material

Additional file 1: Figure S1 Cell morphology of *S. cerevisiae* BY4741 cells upon exposure to 2% C9. C9-treated cells were collected for FESEM (Field Emission Scanning Electron Microscopy) analysis after exposure to C9 for 48 h. For sample preparation, cells were washed with Tris.Cl (pH 7.4) immediately after alkane treatment, and then fixed in 2% glutaraldehyde at 4°C for overnight followed by 1% osmium tetroxide for 10 min. Cell samples were dehydrated through ethanol: 2 min in each of 30%, 50%, 70%, 95% (twice) and 100% (three times). Thereafter, cells were loaded onto silicon slides and dried. Then, samples were coated with platinum and observed using FESEM JSM-6700 F (JEOL). Control, *S. cerevisiae* BY4741 cells in alkane-free medium. Click here for file

Additional file 2: Figure S2 GC chromatograms of alkanes extracted from *S. cerevisiae* BY4741 cells. Control, cells without alkanes, IS, internal standard, C13, n-tridecane. Peaks were indicated by arrows. Click here for file

Additional file 3: Table S1 List of genes differentially regulated in *S. cerevisiae* upon exposure to alkanes. This table contains 7 sub-tables (A-G): A. Genes differently regulated upon exposure to C10 (24 h, and 48 h) and C9 (48 h); B. C9-48 h; C. C10-24 h; D. C10-48 h; E. C11-48 h; F. C12-24 h. G. C12-48 h. **Table S2.** Validation of microarray data by qPCR upon exposure to C10 for 48 h. **Table S3.** ROS levels in *S. cerevisiae* upon exposure to C9 and C10 for 24 h. **Table S4.** Yeast strains and plasmids used in this study. **Table S5.** Primers used in this study. Click here for file

Additional file 4: Figure S3 GC chromatograms of C10 and C11 from *S. cerevisiae* BYL251K cells. IS, internal standard. Peaks were indicated by arrows. Click here for file
