# Supplementary figures and images for: Transcriptome response to alkane biofuels in Saccharomyces cerevisiae: identification of efflux pumps involved in alkane tolerance
Source: Biotechnol Biofuels. 2013 Jul 5;6:95. doi: 10.1186/1754-6834-6-95 (PMC3717029; doi:10.1186/1754-6834-6-95)

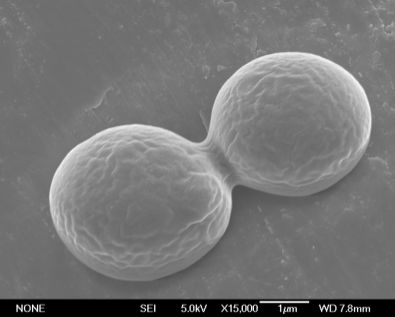

Control

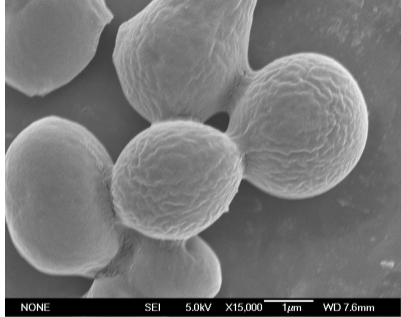

C9

Supplement: Additional file 1: Figure S1 — Cell morphology of S. cerevisiae BY4741 cells upon exposure to 2% C9. C9-treated cells were collected for FESEM (Field Emission Scanning Electron Microscopy) analysis after exposure to C9 for 48 h. For sample preparation, cells were washed with Tris.Cl (pH 7.4) immediately after alkane treatment, and then fixed in 2% glutaraldehyde at 4°C for overnight followed by 1% osmium tetroxide for 10 min. Cell samples were dehydrated through ethanol: 2 min in each of 30%, 50%, 70%, 95% (twice) and 100% (three times). Thereafter, cells were loaded onto silicon slides and dried. Then, samples were coated with platinum and observed using FESEM JSM-6700 F (JEOL). Control, S. cerevisiae BY4741 cells in alkane-free medium. [file 1754-6834-6-95-S1.pdf]

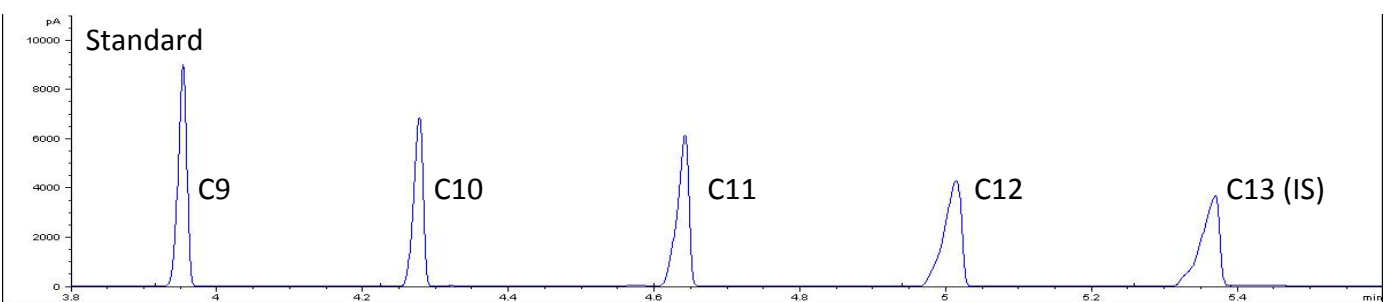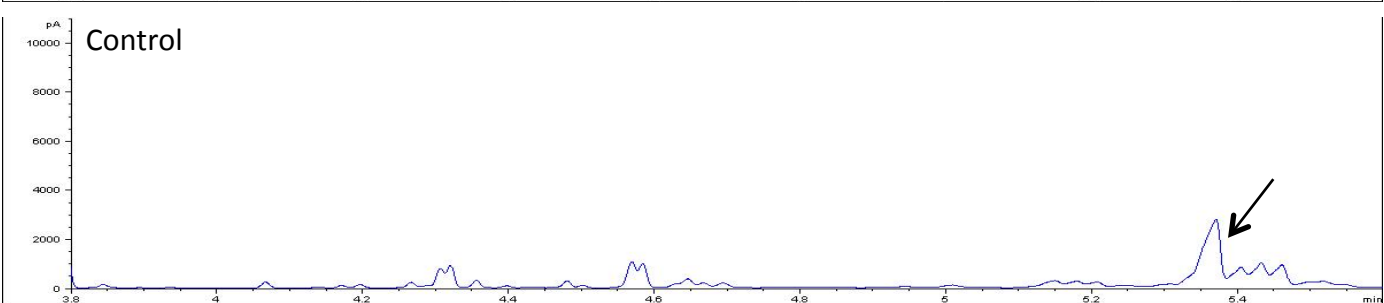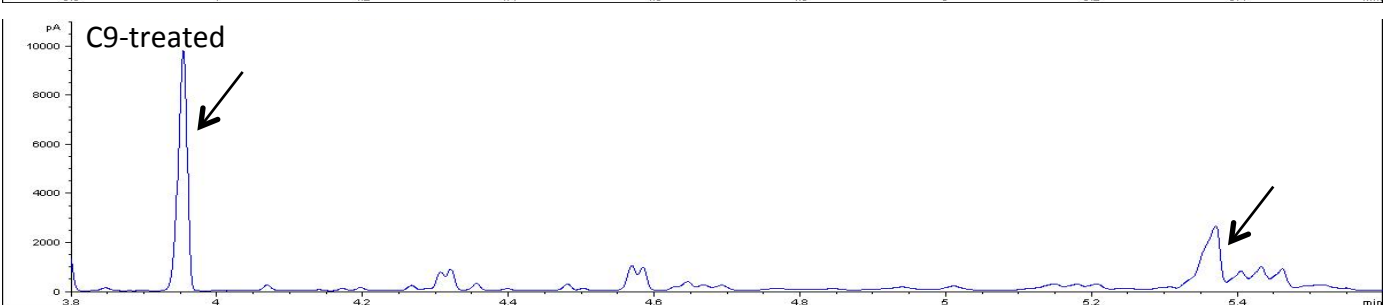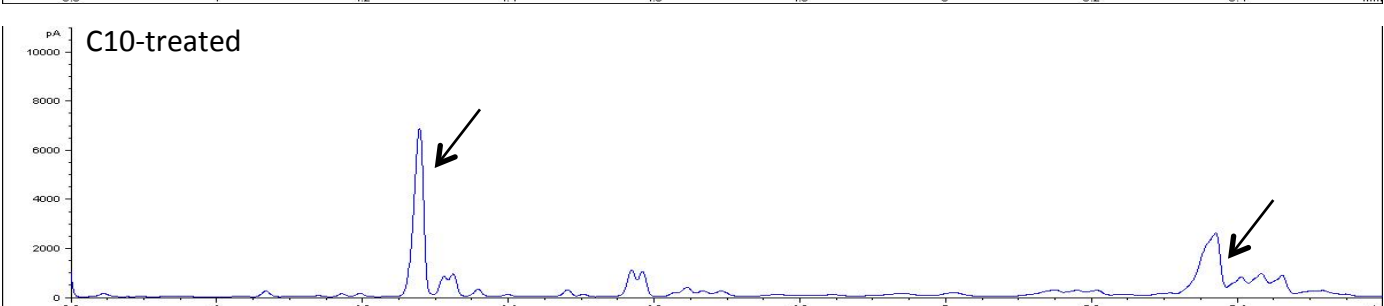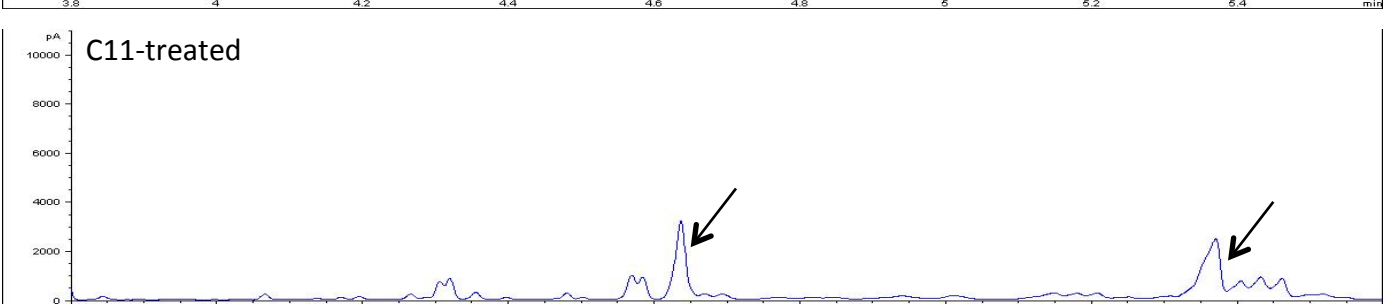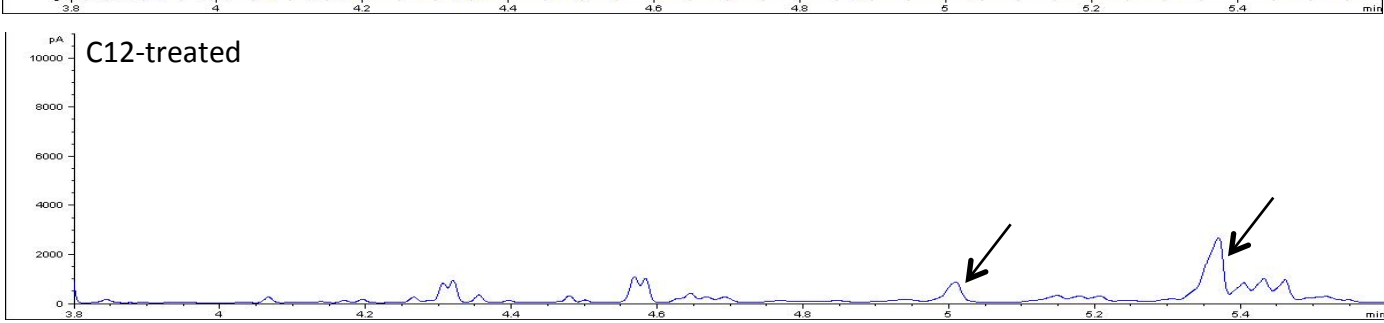

Supplement: Additional file 2: Figure S2 — GC chromatograms of alkanes extracted from S. cerevisiae BY4741 cells. Control, cells without alkanes, IS, internal standard, C13, n-tridecane. Peaks were indicated by arrows. [file 1754-6834-6-95-S2.pdf]

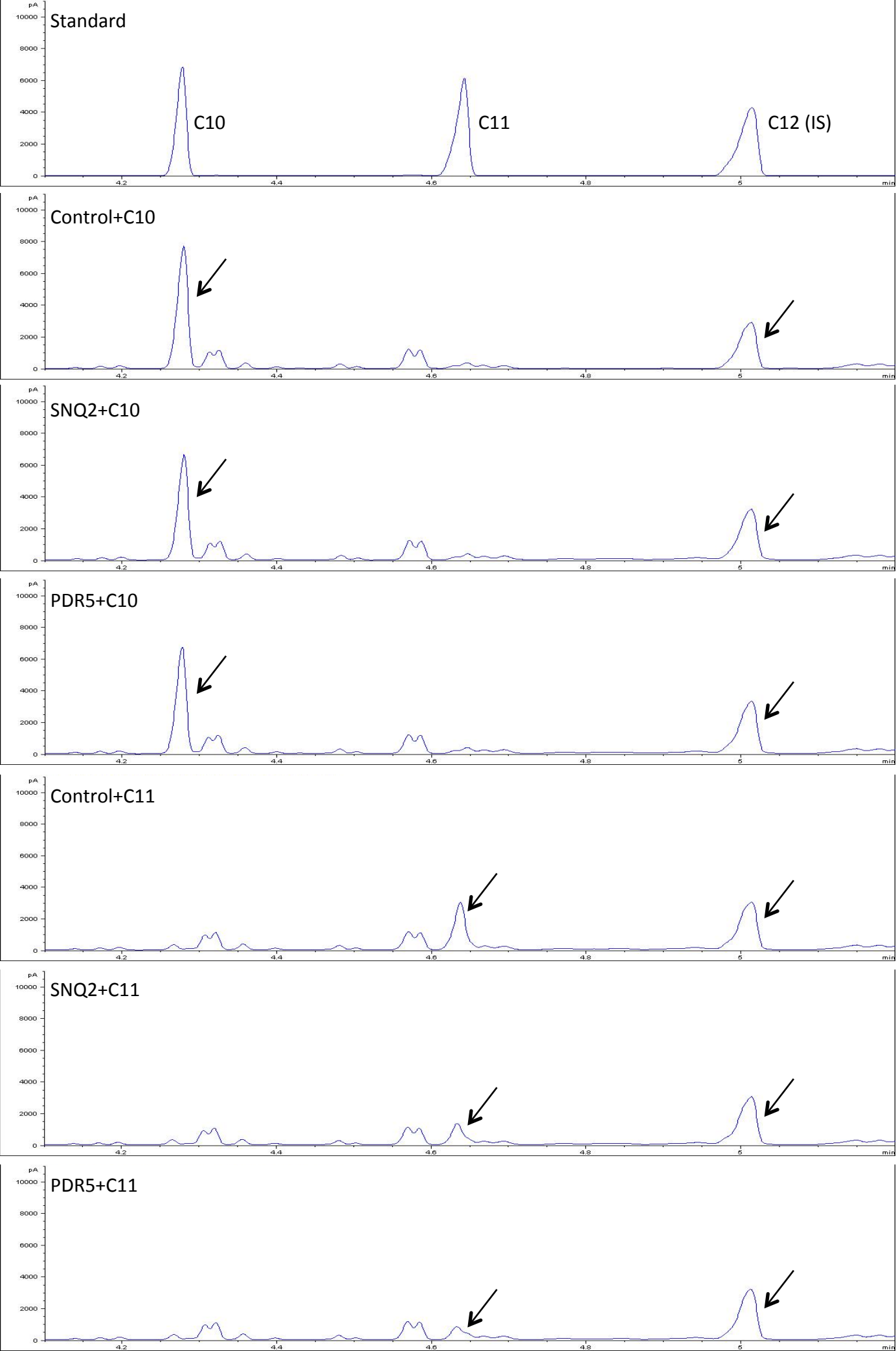

Supplement: Additional file 4: Figure S3 — GC chromatograms of C10 and C11 from S. cerevisiae BYL251K cells. IS, internal standard. Peaks were indicated by arrows. [file 1754-6834-6-95-S4.pdf]
